# Utilizing Mobile Health Technology to Enhance Brace Compliance: Feasibility and Effectiveness of an App-Based Monitoring System for Adolescents with Idiopathic Scoliosis

**DOI:** 10.3390/jpm15090405

**Published:** 2025-09-01

**Authors:** Judith Sánchez-Raya, Judith Salat-Batlle, Diana Castilla, Irene Zaragozá, Azucena García-Palacios, Carlos Suso-Ribera

**Affiliations:** 1 Vall d’Hebron Barelona Hospital Campus, 08035 Barcelona, Spain; judith.sanchez@vallhebron.cat (J.S.-R.); judith.salat@vhir.org (J.S.-B.); 2 Vall d’Hebron Research Institute (VHIR), 08035 Barcelona, Spain; 3 Department of Personality, Evaluation and Psychological Treatment, Universidad de Valencia, 46010 Valencia, Spain; diana.castilla@uv.es (D.C.); irene.zaragoza-alvarez@uv.es (I.Z.); 4 CIBER of Physiopathology of Obesity and Nutrition (CIBERON), 28029 Madrid, Spain; azucena@uji.es; 5 Basic and Clinical Psychology and Psychobiology, Health Sciences, Universitat Jaume I, 12006 Castelló de la Plan, Spain; 6 Department of Psychology and Sociology, Health Sciences, Universidad Europea de Canarias, 38300 La Orotava, Spain

**Keywords:** adolescent idiopathic scoliosis, brace adherence, mobile health (mHealth), patient-reported outcomes, personalized medicine, digital health monitoring

## Abstract

**Background/Objectives:** Adolescent idiopathic scoliosis (AIS) often requires prolonged brace use to prevent curve progression. However, adherence is challenging due to discomfort, mobility restrictions, and psychosocial stressors. This study evaluated the feasibility and clinical utility of a mobile health (mHealth) system for real-time tracking of brace adherence and treatment-related experiences in adolescents with AIS. **Methods:** Thirty adolescents with AIS (mean age = 12.9, SD = 1.8) undergoing brace treatment at a tertiary care center used a custom app for 90 days. The app collected daily self-reports on brace wear duration, discomfort, movement limitations, emotional distress, and social challenges. A clinical alarm system alerted providers when patient input indicated potential concerns. Primary outcomes were feasibility (adherence to daily use and usability ratings) and brace adherence. Secondary outcomes included the app’s capacity to identify treatment-related challenges and its association with changes in stress, quality of life, anxiety, and depression. **Results:** Participants reported meeting recommended brace wear time (≥16 h/day) on 84.8% of days. The app triggered 186 clinical alarms, with the most frequent related to emotional distress (23.1%) and pain (15.6%). Alarm frequency declined over time. Improvements of ≥20% in psychological outcomes were observed in 20–26.7% of participants, while group-level changes were nonsignificant. **Conclusions:** mHealth-based monitoring appears feasible and acceptable for digitally engaged adolescents with AIS. The app supported early detection of treatment barriers and prompted timely clinical responses. Despite limitations, it shows promise as a tool to improve treatment engagement and address psychosocial challenges in scoliosis care.

## 1. Introduction

Adolescent idiopathic scoliosis (AIS) is the most common form of spinal deformity in otherwise healthy adolescents. Bracing is the standard conservative treatment for moderate curves, with proven efficacy in halting progression when adherence is adequate [1,2]. However, many adolescents struggle to comply due to discomfort, restricted mobility, emotional distress, and social stigma, with adherence rates often falling short of clinical recommendations [3,4].

Monitoring adherence is crucial but traditionally relies on self-reports or diaries, which are vulnerable to recall bias and often omit relevant psychosocial factors such as pain, mood, and body image concerns [5,6]. Although embedded sensors objectively track brace wear [7,8,9], they do not capture the subjective experiences that often drive non-compliance [10,11]. Thus, there is a growing need for holistic monitoring approaches that integrate patient-reported outcomes to inform timely, individualized care.

Mobile health (mHealth) technologies, particularly smartphone applications (apps), are usually well-accepted tools [12] and offer a promising means of capturing both adherence behavior and psychosocial factors in real time [13,14]. With over 80% of adolescents owning smartphones, these devices provide an accessible and convenient platform for ecological momentary assessment of brace adherence and associated biopsychosocial factors [15,16,17,18].

Building on this potential of mHealth, we adapted the Pain Monitor platform—originally developed for chronic pain patients [19]—to develop an app-based system for adolescents undergoing brace treatment for AIS. The system collects daily ecological momentary assessments of brace use, physical symptoms, emotional distress, and social challenges, and includes a clinical alarm function that notifies providers of patient-reported concerns [20].

Although digital tools are increasingly used in adolescent health, their integration into AIS treatment is still limited [13]. Therefore, his study aimed to evaluate the feasibility, usability, and clinical utility of this system over a 90-day period. Specifically, we examined adolescents’ engagement with the app, its ability to detect adherence-related and psychosocial difficulties, and its potential to support individualized, responsive care in the management of AIS.

## 2. Materials and Methods

### 2.1. Design and Participants

This study employed a single-group, open-trial design to evaluate the feasibility of Pain Monitor version 2.0 (**©** Jaume I University, Castelló, Spain), an app-based monitoring system for brace compliance in adolescents with idiopathic scoliosis (IS). Participants were recruited from the Physical Medicine and Rehabilitation Service at Vall d’Hebron University Hospital, a tertiary care center in Spain.

Eligible participants were adolescents aged 10 to 18 years with primary spinal curvatures between 25° and 40° who were initiating brace treatment. All participants were prescribed full-day use (18–23 h) of the same type of rigid thoracolumbosacral orthosis, typically used for AIS, in accordance with SOSORT guidelines [8]. Inclusion criteria required that participants had no prior experience with brace use, owned a smartphone (Android or iOS), and possessed the cognitive and physical capacity to engage with the mobile app. Both participants and their legal guardians provided informed consent before enrollment.

Given the novelty of the technological component and its current absence from routine clinical care, a small-scale study was deemed appropriate as a preliminary step prior to larger-scale implementation, in line with established recommendations [21]. A sample size of approximately 30 participants is commonly considered sufficient in feasibility studies to assess key parameters such as adherence, user satisfaction, and usability [22], and is consistent with prior research involving novel monitoring technologies in medical settings [23].

### 2.2. Procedure

Eligible patients were informed about the study by their medical team prior to initiating brace treatment. Those who agreed to participate signed an informed consent form along with their parents. Participants then provided their email addresses and completed a baseline assessment online via the Qualtrics platform. To ensure anonymity, each participant was assigned a unique alphanumeric code, which was used to anonymize responses in both the app and survey data.

During their medical appointment, participants were guided through the installation and setup of the Pain Monitor app, which had been adapted for IS management. The app was available for free download via the Google Play Store. Research staff provided hands-on training to ensure familiarity with the app’s functions.

Participants were instructed to use the app daily for 90 days, completing brief assessments between 7 p.m. and 9 p.m. These assessments tracked brace wear duration and associated experiences, including physical symptoms (e.g., pain, movement restriction) and psychosocial factors (e.g., emotional distress, social challenges). Each daily self-report in the app was designed to take approximately 2–3 min to complete.

In addition to daily monitoring, the app generated clinical alarms to alert healthcare providers to potential issues, which were reviewed the following morning by the clinical team.

At the end of the 90-day period, participants completed a post-treatment assessment via Qualtrics to evaluate usability and adherence. The study was approved by the Ethics Committee of Vall d’Hebron University Hospital and registered at ClinicalTrials.gov (NCT04881591).

### 2.3. App Development and Functioning and Clinical Alarms

The Pain Monitor app was originally designed for chronic pain patients [19] and was adapted for AIS management by a multidisciplinary team. The app allowed real-time reporting of brace use and treatment-related experiences, facilitating ecological momentary assessments in a natural setting.

A clinical alarm system was integrated to notify physicians of significant concerns, such as persistent pain, emotional distress, or adherence issues. Alarms were automatically sent via email to the medical team at 7 a.m. the following morning. The app did not provide immediate feedback to patients to prevent the expectation of real-time clinical intervention. Instead, concerns were addressed during subsequent consultations, with urgent matters requiring direct contact with the medical team.

Daily app items were based on validated questionnaires and assessed brace adherence (hours of wear), physical symptoms (pain from pressure or irritation, movement restrictions), psychosocial factors (teasing, emotional distress, social support), and daily interference (impact on sleep, activities, mood).

A detailed description of the app’s daily assessments and alarm triggers is available in the study protocol [14].

### 2.4. Outcome Measures

#### 2.4.1. Primary Outcomes: Feasibility and Acceptability

Feasibility was assessed using both objective and subjective measures of acceptability. Objective acceptability included adherence to app use, measured as the proportion of completed daily assessments over 90 days. Subjective acceptability was evaluated with the System Usability Scale (SUS), recently adapted to Spanish [24]. The SUS consists of 10 items rated on a 5-point Likert scale, with total scores ranging from 0 to 100. Higher scores indicate better usability [25].

Study reach was also evaluated, defined as the percentage of eligible participants who successfully installed and used the app.

#### 2.4.2. Secondary Outcomes: Clinical Utility and Effectiveness

App utility was measured through participant-reported adverse effects (e.g., pain, discomfort, emotional distress), frequency of triggered clinical alarms, and provider responses.

Self-reported brace adherence was assessed with daily logs of brace wear duration, categorized as full-time (≥16 h), partial adherence (8–16 h), or non-adherence (<8 h), following guidelines [8]. Brace wear was reported in distinct time blocks: morning (wake-up to midday), evening (midday to bedtime), and sleeping (during the night). Participants could report multiple blocks per day, allowing for flexible wear schedules.

Preliminary effectiveness included an evaluation of changes in stress, quality of life, anxiety, and depression from baseline (before brace use initiation and prior to any app-based monitoring) to post-treatment (90 days), measured using the following:

1. Sobberheim Stress Questionnaire (BSSQ; Spanish adaptation) [26,27]: An 8-item measure of psychological stress caused by brace use in AIS patients. Higher scores reflect less stress.
2. Italian Spine Youth Quality of Life Questionnaire (ISYQOL; Spanish adaptation) [28,29]: A tool measuring health-related quality of life in relation to back problems. Higher scores reflect poorer health status.
3. Scoliosis Research Society-22 Questionnaire (SRS-22; Spanish version) [30,31]: A 22-item scale assessing perceived quality of life. Higher scores indicate better quality of life.
4. Hospital Anxiety and Depression Scale (HADS; Spanish validation) [32,33]: A 14-item measure of anxiety and depressive symptoms. Higher scores reflect more anxiety and depression.
5. Trunk Appearance Perception Scale (TAPS) [34]: A tool for assessing the subjective perception of trunk deformity in patients with idiopathic scoliosis, using a visual scale with three images. Higher scores reflect a more severe perception of trunk deformity.

Internal consistency (Cronbach’s alpha) for the measures in the current sample was 0.84 (Sobberheim Stress Questionnaire), 0.86 (Italian Spine Youth Quality of Life Questionnaire), 0.68 (Scoliosis Research Society-22 Questionnaire), and 0.87 and 0.62 for the anxiety and depression subscales of the Hospital Anxiety and Depression Scale, respectively.

### 2.5. Analyses

The analysis of feasibility and clinical utility outcomes was primarily descriptive. Adherence to daily app-based monitoring was evaluated by calculating the percentage of completed assessments over the total expected entries during the 90-day study period. Means, standard deviations, and response rate trends across the three months were examined to assess fluctuations in engagement. Usability ratings, as measured by the System Usability Scale (SUS), were summarized using mean scores and standard deviations. The reach of the intervention, defined as the percentage of eligible participants who successfully installed and used the app, was also calculated.

To assess the app’s utility in detecting adverse effects and adherence barriers, we analyzed the frequency and distribution of reported brace-related challenges, including pain, movement restrictions, emotional distress, and social difficulties. The total number of clinical alarms generated throughout the study was documented, and trends in alarm frequency were examined across the three-month period. Additionally, provider responses to alarms were categorized based on the type of intervention (e.g., email support, phone call, adjusted medical appointment), and response patterns were analyzed to determine their effectiveness in addressing reported issues.

Self-reported brace adherence was analyzed by calculating the proportion of days participants met full-time wear (≥16 h), partial adherence (8–16 h), or non-adherence (<8 h). Trends in brace wear patterns were examined over the three months to determine whether adherence improved or declined over time. Additionally, the relationship between adherence and reported adverse effects was explored to assess whether discomfort or psychosocial factors influenced compliance.

Preliminary effectiveness analyses were conducted at both the individual and group levels, as recommended for research involving small sample sizes [35].

Individual-level changes in clinical outcomes were assessed using the Individual Percentage of Improvement (IPI) method, which calculates the percentage change in each participant’s score from baseline to post-treatment [36]. A threshold of 20% improvement was used to determine meaningful clinical change. This approach was selected based on prior research using similar cut-offs in short-term psychological interventions and chronic disease management [37,38].

For group-level comparisons, changes in stress, quality of life, anxiety, and depression scores from baseline to the three-month follow-up were analyzed. Given the small sample size and the non-normal distribution of some variables, non-parametric Wilcoxon signed-rank tests were performed to compare pre- and post-treatment outcomes. This test was chosen due to its suitability for paired data in small-sample studies with potential violations of normality assumptions. Statistical significance was set at *p* < 0.05, and all analyses were conducted using SPSS version 28 [39].

## 3. Results

### 3.1. Feasibility and Acceptability

A total of 44 eligible patients were invited to participate, of whom 38 (86.4%) completed the baseline assessment and downloaded the app, demonstrating a high initial reach. However, eight participants (21.1%) discontinued the study within the first week, citing lack of interest in external monitoring. The final sample consisted of 30 adolescents (mean age = 12.9 years, SD = 1.8), all of whom completed the 90-day monitoring period and the follow-up assessment.

Objective acceptability, measured by adherence to app use, indicated that participants completed daily assessments on 69.9% of study days (range: 21–86 assessments per participant). Adherence rates declined over time, with response rates of 75.2% in the first month, 66.0% in the second month, and 58.0% in the third month.

Subjective acceptability, assessed via the System Usability Scale (SUS), yielded an initial mean score of 82.3 (SD = 12.4, range: 50.0–97.5), indicating excellent usability. At the end of the study, the mean SUS score increased to 86.3 (SD = 9.7, range: 65.0–100), suggesting sustained high satisfaction with the app.

### 3.2. App Utility and Clinical Alarms

Over the three-month duration of app usage, 151 clinical alarms were reported and automatically transmitted to the medical team for review.

These alarms comprised a variety of patient-reported experiences (Table 1). The most frequently reported types included pain-related alarms, all attributable to brace wear, which collectively constituted 31.79% of the total clinical alarms (primarily persistent pain, reported as greater than 2 for 3 consecutive days, at 16.56% and pain specifically from brace pressure at 15.23%). Discomfort interfering with sleep was also a prominent concern (17.88%).

Other significant brace-related alarms, each representing 5.96%, included general discomfort, intense overwhelming feeling/distress, and intense discomfort interfering with body image/perception. Remaining alarms (each under 5%) encompassed issues such as pain from rubbing/chafing, intense discomfort affecting clothing choice and style, feeling excessive heat, intense embarrassment/social unease, and various emotional states like anxiety, frustration, intense mood interference, anger, and sadness.

Despite being designed to monitor a comprehensive range of potential challenges, several specific alarm categories were not triggered by any participant throughout the study duration: lack of perceived social support, social teasing, difficulties in discussing brace wear or interference with daily activities (e.g., significant disruption to academic pursuits, interactions with friends and family, or leisure activities).

Beyond clinical alarms, the app was also designed to generate a specific non-clinical alert: a ‘non-entry’ alarm. This alarm was triggered when a participant failed to respond to the app’s scheduled questions for two consecutive days. Every participant in the study experienced at least one instance of this ‘non-entry’ alarm. Notably, for 48.7% of participants, these ‘non-entry’ alarms were the sole type of alert received, with no clinical alarms recorded for them.

Regarding the types of response from the medical team, of the total alarms recorded 50.6% did not necessitate a direct response. These were managed through a wait-and-see approach following initial review, with intervention deferred until the next response. Conversely, email communication was employed for 38.5% of alarms with content tailored to the alert type (e.g., inquiring about app issues for ‘non-entry’ alarms or detailing pain for ‘brace pressure’ alarms). When email was unfeasible, direct phone calls were made, accounting for 4.5% of alarms.

Following initial contact via email or phone call, only seven patients continued to experience persistent alarms that required further intervention. For these cases, the response was escalated: three patients received a specially prepared support document from the research team. This document, developed by a team of four psychologists, was designed to provide psychological support in the absence of an in-unit psychologist when alarms associated with psychological distress were received. For the remaining four patients, an earlier clinic visit was proposed to the patient/family.

In terms of the medical team’s response time, a systematic approach guided the management of alarms. A substantial 52.36% of all alarms were handled by deferring an immediate intervention. This percentage encompasses the 50.6% where a non-immediate response was a deliberate decision by the medical team, and an additional 1.76%, despite requiring direct intervention, could not be addressed promptly due to medical staff holidays. Concerning the speed of response for alarms necessitating direct intervention, 18.90% were addressed on the same day (0 days). Subsequently, 14.56% of alarms were addressed within 1 day, 8.13% within 2 days, and 3.59% within 3 days. The remaining 2.46% of alarms were addressed within 4 to 6 days.

### 3.3. Brace Adherence

Self-reported brace adherence indicated that participants met the recommended 16 h daily wear time on 84.8% of study days. Full-time (20 to 24 h) adherence was reported on 53.6% of days, while non-adherence (brace use < 8 h) was rare, occurring on 2.2% of days (Table 2).

Adherence slightly improved over time, increasing from 50.3% in the first month to 55.7% in the third month. Sixteen adherence-related clinical alarms were recorded, with most occurring in the first month (50%), followed by the second month (25%) and third month (12.5%).

### 3.4. Changes in Clinical Outcomes

At the individual level (Table 3), at least 20% improvement was observed in stress, health-related quality of life, anxiety, and depression for 20–26.7% of participants, while only 6.7% and 8.8% showed similar improvement in overall quality of life and perceived trunk deformity. Deterioration of ≥20% was rare, except for health-related quality of life, where 23.3% of participants reported a decline.

At the group level (Table 4), paired samples *t*-tests showed no statistically significant changes in any clinical outcomes from baseline to post-treatment:

- Stress (BSSQ): t(29) = −0.39, *p* = 0.698.
- General Quality of Life (SRS-22): t(29) = −1.15, *p* = 0.261.
- Health-Related Quality of Life (ISYQOL): t(29) = −0.09, *p* = 0.931.
- Anxiety (HADS-A): t(29) = 1.78, *p* = 0.086.
- Depression (HADS-D): t(29) = 1.46, *p* = 0.157.
- Perceived deformity (TAPS): t(29) = 0.83, *p* = 0.413.

## 4. Discussion

This study evaluated the feasibility and clinical utility of a smartphone-based monitoring system for brace compliance and associated biopsychosocial factors in adolescents with IS. Our findings suggest that app-based monitoring can be a practical adjunct to traditional care, enabling real-time tracking of treatment adherence and psychosocial responses. However, key limitations and potential biases must be considered in interpreting the results.

### 4.1. Feasibility and Acceptability of the App

App feasibility was moderate to high. Of the 44 patients invited, 86.4% enrolled and initiated app use—an encouraging indication of initial acceptability among adolescents. However, 21.1% discontinued use within the first week, largely citing lack of interest. These results are consistent with previous findings showing very high attrition rates in adolescent mHealth interventions, especially during the first days of use. For instance, over 95% of participants stopped using the app within 12 weeks in one trial, with 44% abandoning it during the first week [40]. Similar patterns were observed in obesity interventions, where more than half of adolescents in outpatient care used the app for less than a week [5]. Time-specific usage analyses from another study also revealed steep declines in engagement after the first week, with attrition starting as early as week three [41]. These converging findings highlight the importance of designing adolescent mHealth tools that can sustain attention and motivation beyond initial novelty.

Among those who remained in the study, daily engagement rates (69.9%) were comparable to similar studies of adolescent digital health interventions and showed a gradual but expected decline over time [42].

The app’s usability was rated as excellent (mean SUS = 86.3), with improved ratings at follow-up. These results align with prior findings that adolescents generally report high usability and acceptance of well-designed mobile tools [43,44]. Still, as noted in other digital interventions [45], high usability does not guarantee long-term engagement or efficacy.

### 4.2. Brace Adherence and Treatment Engagement

Self-reported brace adherence was generally high, with participants meeting the recommended 16 h daily wear time on 84.8% of days. Full-time (24 h) adherence was reported on 53.6% of days, and non-adherence was rare (2.2% of days). These findings suggest high adherence, given the recommended threshold of over 80% of prescribed daily use [46] and exceed averages reported in the literature [3], which may be partially attributed to the structured daily monitoring and enhanced sense of accountability created by the app. This aligns with evidence suggesting that routine monitoring can improve treatment engagement [47].

Interestingly, full-time adherence increased slightly over the 90-day period, supporting the hypothesis that adaptation to brace treatment improves over time. Notably, only 1.6% of all clinical alarms were related to adherence concerns—suggesting high compliance within this engaged sample.

However, it must be acknowledged that brace adherence was self-reported, which may overestimate actual wear time. Unlike embedded sensors, the app cannot provide precise hour-by-hour tracking. It relies on structured self-report entries covering time blocks (e.g., morning, evening, and sleeping) rather than exact duration. Thus, although the results are promising, they must be interpreted cautiously. Future studies should incorporate objective adherence sensors to validate these findings.

Still, the app may have served a valuable supportive role. Prior research shows that adolescents undergoing brace treatment often feel isolated or unsupported [48]. By enabling structured reflection and facilitating clinical communication, the app may have helped counteract this dynamic, contributing to sustained engagement. The fact that 80% of participants who initiated app use completed the full 90-day protocol underscores its potential to support treatment continuity, especially considering that maintaining adolescent engagement in health interventions is often a challenge [17]. Future studies could explore additional strategies to further enhance engagement, such as gamification, personalized feedback, or integrated interventions within the app [49].

### 4.3. App Utility and Clinical Alarms

The app successfully captured real-time patient-reported experiences, allowing for early identification of adherence barriers and adverse effects. By detecting concerns such as brace-related pain, intense discomfort interfering with sleep or body image, or even the lack of data entry, the system provided a proactive layer of patient support. This enabled the medical team to implement early interventions—such as physician outreach (via email or phone), educational resources (like the support document), or self-management strategies—which successfully resolved many initial challenges. The effectiveness of this approach is evidenced by the fact that only seven patients continued to experience persistent alarms requiring further escalated intervention, leading to specialized psychological support or an earlier clinic visit. This highlights the app’s potential to facilitate prompt, less intensive responses, preventing minor issues from escalating and ultimately supporting adherence more effectively than traditional methods.

Our app’s real-time data capture and alarm notification system align with this evidence, demonstrating how digital tools can enhance clinical responsiveness and patient-centered care.

The app’s clinical utility was evident in its ability to detect a wide range of patient-reported challenges and to trigger automated alerts that informed clinical decision-making. Across the study period, most alerts were not related to brace wear time but to psychosocial and physical symptoms—such as pain, sleep disruption, and emotional distress.

Importantly, emotional and psychosocial distress alarms accounted for 40.7% of clinical alerts, surpassing pain-related alarms (31.8%). This reinforces the role of emotional and cognitive factors—such as body image concerns, stigma, and distress—as key influences on adherence [50,51]. While pain is an important consideration, these findings suggest that emotional burden may be even more central in shaping the adolescent experience of brace treatment. The app’s ability to surface these issues in real time represents a major strength.

Clinical responses to alarms were generally swift and proportionate, ranging from email check-ins to early clinic appointments. Only seven participants required escalated intervention, suggesting that early, light-touch actions (e.g., support documents and phone calls) were often sufficient to resolve concerns. This layered response model supports the feasibility of app-driven, stepped-care approaches in pediatric rehabilitation and the importance of efficient communication and timely information exchange in improving patient outcomes [52].

Interestingly, several expected categories of distress—such as social teasing or peer-related stigma—did not trigger alarms. This absence may reflect limitations in item sensitivity or reluctance to report such experiences. Alternatively, it may relate to sample characteristics or timing of data collection. Further refinement of app content and prompts could improve the system’s ability to capture more nuanced social difficulties.

The high prevalence of ‘non-entry’ alarms, particularly in the latter months, underscores the importance of monitoring engagement alongside clinical symptoms. These passive indicators may offer early warning signs of dropout risk or reduced motivation—signals that warrant proactive follow-up.

### 4.4. Clinical Outcomes and Patient Well-Being

Although the primary aim of this study was feasibility, we explored preliminary changes in psychological and quality-of-life outcomes. At the individual level, 20–26.7% of participants reported ≥20% improvement in stress, health-related quality of life, anxiety, or depression—suggesting that some adolescents perceived subjective benefits. However, this should not be interpreted as evidence of efficacy, as the study was not designed or powered to evaluate psychological change, and no control group was included.

At the group level, no statistically significant improvements were found, which is expected in a feasibility study. Importantly, there was no widespread deterioration over time—an encouraging finding, considering that brace treatment is often associated with worsening psychosocial outcomes [48]. The structured monitoring and regular contact may have offered participants a sense of support, but definitive conclusions about mental health benefits cannot be drawn from this study.

Future research should include randomized controlled designs and embedded psychological interventions to examine whether such apps can improve psychological outcomes in a clinically meaningful way.

### 4.5. Limitations and Future Directions

This study has several limitations. First, the sample was relatively small and likely skewed toward digitally engaged participants, limiting generalizability. Nearly one in five participants discontinued app use in the first week, which may reflect a self-selection bias: those more motivated or comfortable with technology may have been overrepresented. This pattern has been observed in other adolescent mHealth studies [5], where early attrition is common and associated with lower motivation or engagement. Thus, findings related to satisfaction and usability may not generalize to all brace-wearing adolescents.

Second, all outcome measures—including brace use and emotional states—were self-reported, which introduces the risk of response bias. The inclusion of objective adherence sensors and clinician-rated outcomes in future studies is strongly recommended. Third, while the app identified emotional distress, it did not include embedded psychological interventions. Given the frequency and relevance of emotional challenges observed, integrating in-app support (e.g., cognitive behavioral therapy modules, mood trackers, or peer support) may enhance future versions.

Finally, the short study duration limits insights into long-term effects. Ongoing studies should explore whether the benefits of digital monitoring persist and translate into improved clinical outcomes.

### 4.6. Conclusions

This study provides preliminary evidence that smartphone-based monitoring is a feasible, acceptable, and potentially valuable tool for supporting brace adherence and addressing biopsychosocial needs in adolescents with idiopathic scoliosis. The app’s usability was high, and its alarm system enabled timely, proportionate responses to emerging concerns. Emotional distress emerged as a more frequent alarm trigger than physical pain, underscoring the central role of psychosocial support in brace management.

Digital tools such as this may help bridge the gap between adolescent patients and clinical teams by enabling real-time communication, personalizing care, and improving treatment adherence. As part of a broader care model, app-based monitoring systems could enhance multidisciplinary coordination and patient outcomes. Future research should prioritize randomized controlled trials, integration of objective monitoring, and development of embedded psychological interventions to maximize clinical impact.

## Figures and Tables

**Table 1:** Clinical alarms received through daily reports in the app.

Alarm Associated with Brace Use	Frequency (%)
Pain ≥ 3 (3 consecutive days)	29 (15.6%)
Interference with sleep (3 consecutive days)	30 (16.1%)
Interference with body image/clothing (5 consecutive days)	21 (11.3%)
General discomfort	12 (6.5%)
Very poor adherence (<8 h daily; 7 consecutive days)	13 (7.0%)
Poor adherence (8–16 h daily; 15 consecutive days)	3 (1.6%)
Frustration or emotional burden (i.e., shame, depression, anxiety, or anger; 5 consecutive days)	43 (23.1%)
Interference with going out (3 consecutive days)	4 (2.2%)
Moving difficulties (3 consecutive days)	12 (6.5%)
Excessive sweating (7 consecutive days)	19 (10.2%)

**Table 2:** Daily self-reported brace use.

Brace Use	Month 1	Month 2	Month 3	Total
Never	3.7	1.5	2.4	2.2
8 h	14.7	9.6	13.5	14.8
Morning	1.0	0.0	0.7	0.6
Evening	6.4	0.0	1.7	3.0
Sleeping	7.3	9.6	11.8	9.3
16 h	31.3	34.8	27.7	31.2
Morning + evening	3.6	3.9	0.0	2.5
Morning + sleeping	7.5	9.8	12.8	9.6
Evening + sleeping	20.3	21.1	14.9	19.1
24 h	50.3	54.1	55.7	53.6

Brace wear was self-reported using predefined time blocks: morning (wake-up to midday), evening (midday to bedtime) and sleeping (during the night). Participants could select one or more time blocks per day.

**Table 3:** Changes in clinical outcomes at the individual level.

	≥20% Improvement	<20% Improvement	No Change	<20% Deterioration	≥20% Deterioration
Stress (BSSQ)	20.0%	26.7%	16.7%	26.7%	10.0%
Quality of life (SRS)	6.7%	50.0%	6.7%	33.3%	3.3%
Health-related Quality of life (ISYQOL)	20.0%	30.0%	10.0%	16.7%	23.3%
Anxiety (HADS)	26.7%	6.7%	26.7%	13.3%	6.7%
Depression (HADS)	20.0%	20.0%	26.7%	16.7%	10.0%
Appearance (TAPS)	8.8%	35.3%	35.3%	14.8%	5.9%

Numbers indicate the percentage of participants experiencing that level of change. BSSQ, Bad Sobernheim Stress Questionnaire; SRS, Scoliosis Research Society Questionnaire; ISYQOL, Spine Youth Quality of Life questionnaire; HADS, Hospital Anxiety and Depression Scales, TAPS, Trunk Appearance Perception Scale.

**Table 4:** Changes in clinical outcomes at the group level (Wilconxon’s test).

	Pre-Treatment Mean (SD)	Post-Treatment Mean (SD)	Z	*p*
Stress (BSSQ)	21.8 (4.7)	22.1 (4.5)	−0.56	0.579
Quality of life (SRS)	81.9 (10.4)	83.4 (8.9)	−1.04	0.299
Health-related Quality of life (ISYQOL)	13.5 (5.3)	13.6 (5.9)	−0.11	0.914
Anxiety (HADS)	11.3 (4.0)	10.3 (3.7)	−1.54	0.124
Depression (HADS)	9.1 (2.0)	8.6 (2.0)	−1.30	0.195

BSSQ, Bad Sobernheim Stress Questionnaire; SRS, Scoliosis Research Society Questionnaire; ISYQOL, Spine Youth Quality of Life questionnaire; HADS, Hospital Anxiety and Depression Scales.

## Data Availability

Data will be shared upon reasonable request.
